# Transforming Integration through General Practice: Learning from a UK Primary Care Improvement Programme

**DOI:** 10.5334/ijic.3044

**Published:** 2018-05-18

**Authors:** Robin Miller

**Affiliations:** 1 Health Services Management Centre, University of Birmingham, Park House, Edgbaston Park Road, Birmingham B15 2RT, UK

**Keywords:** general practice, primary care, transformation, commissioning, integration

## Abstract

This article addresses the challenge of how to implement integration within primary care services. It shares learning from a UK based improvement programme which reflected international interest in transferring activities from hospital and community and developing holistic primary care that responds to the needs of the local community. Programme components included additional per capita funding for involved practices, monthly learning sets between pilot leads, and a formative evaluation. Practices had flexibility in how to use the additional funding to meet local needs and were selected through a competitive process. The programme successfully delivered diagnostic and treatment activities previously provided in acute hospital. Some practices also introduces new holistic approaches which were mostly sustained at the end of the twelve month period. The programme demonstrates that transformation of primary care requires a change in the internal paradigms held by clinicians and purchasers, careful design of learning opportunities, responding to multiple levels of motivation, and deployment of relevant change infrastructures and improvement methodologies.

## Introduction and national context

Primary care is a key component of a more integrated and person-centred health and care system [1,2,3]. English primary care is based around general practice and reflects characteristics reflecting strong primary care – is it largely free at the point of access, supports individuals from ‘cradle to grave’, acts as a co-ordinating point of the wider public health system, and has a capitated budget to support an identified patient population [4,5,6]. Despite these strengths, English general practice and therefore the primary care system in which it is situated, does not consistently demonstrated integrated and person-centred care [7,8,9,10]. This is partly due to structural factors such as conflicting organisational objectives, sectorial policy priorities, and activity based incentive systems. Practice issues related to professional differences, insufficient collaborative skills and lack of system knowledge also contribute [9,10]. National policy priorities for improvement have therefore included co-ordinating care for those with complex needs, encouraging healthy lifestyles, and pro-actively detecting and responding to key long-term conditions [11].

English general practitioners (GPs) have traditionally owned the general practices in which they work and employed reception, practice management and nursing staff members. GPs have a hybrid status that is unusual in the UK NHS as it combines private profit with public sector benefits [12]. This means that GPs are generally not directly managed by government and instead policy makers have to deploy other levers. Responsibility for overseeing general practice has been split between national government and local healthcare purchasers (or *commissioners* in English terminology). Levers to influence the practice of GPs include national contracts setting out expected activities, local incentive payments to encourage adoption of identified health promotion practices, increased diversification of providers to enhance support for under-doctored localities and populations, and independent inspections of general practice by the quality regulator [13,14]. Since 2004 the national Quality and Outcomes Framework has been central to the policy maker – general practice relationship. Initially this pay for performance scheme led to improvement health outcomes and decreased hospital attendance for targeted conditions, but these have largely returned to the previous rate of improvement [15,16]. Doctors and nurses report decrease in person-centred practice since QOF was introduced and that patients are less satisfied with continuity of care [17].

National policy has sought to engage GPs in leading more fundamental reform of the public healthcare system. This includes the transfer of resources from acute to community settings, better horizontal and vertical integration, and more proactive and preventative models of care. Connected national initiatives include general practices holding purchasing budgets (GP Fundholding), primary care led organisations purchasing and providing community health services (Primary Care Trusts) and GP-member organisations commissioning most local health services (Clinical Commissioning Groups) [18]. These have at best been partially successful in either changing local healthcare systems or engaging GPs within the reform process [19,20]. The current drive follows the international trend towards ‘accountable care’ and is encouraging GPs to participate in partnerships with other providers to provide more personalised and co-ordinated care for populations of 30,000 to 50,000 [21,22]. New contractual options which incentivise such partnerships to share financial risks and achieve better inter-professional collaboration are being introduced. The practicalities of implementation are being explored through a series of national programmes. These include the Integrated Care and Support Pioneers (health and social care integration), the New Models of Care programme (acute-community, health-social care, and intensive support around care homes), and the Primary Care Home programme (developing care communities across health and social care) [23].

Whilst general practice is not the only the service of relevance, it is seen as a vital ingredient in these new integrated models of care. Securing the commitment of GPs is therefore essential but experience in the UK and international suggests that engaging these clinicians in reform of primary care be difficult [24,25]. This article reports on an English improvement programme which sought to encourage GPs to develop pilots to deliver more integrated care. Learning from the pilots would then be used as the basis for a large scale transformation programme (i.e. “coordinated, system wide change affecting multiple organizations and care providers” *(p422)* [26]. In particular the article considers the following issue: ‘*how can GPs be motivated and supported to engage in transformation programmes which seek to deliver more integrated care*’?

## Local Context and Improvement Programme

Birmingham Cross-City CCG had commissioning responsibility for a population of approximately 700,000 patients. This population had a diverse socio-economic profile with a substantial representation of minority ethnic communities. The CCG was governed through GP membership model overseen by a Board composed of clinicians, managers and key external stakeholders. The CCG had over a 100 member general practices and an annual budget of £900 million. It faced demographic pressures connected with an ageing population, increasing numbers of people with multiple long-term conditions, and rising rates of mental illness. There were concerns regarding performance in respect of key health outcomes (see **Box 1**) and significant financial pressures. The CCG was committed to achieving savings of £19 million in 2014/15 and £24 million in 2015/16 through reducing emergency admissions (by 15%) and average length of stay in hospital (to seven days) [27].

Alongside the national QOF contracts, the CCG had previously incentivised practices to deliver additional primary care services through ‘local enhanced services’ (LES) schemes. These were connected to specific conditions, and practices could choose which ones to adopt. As a consequence patients did not all receive a similar offer from all general practices, and the CCG could not decommission services from the acute sector as there was no guarantee that a primary care alternative would be available (see **Box 2**). A further issue were national concerns in England regarding the sustainability and changing nature of the general practice workforce [28].

**Box 1: Concerns regarding health outcomes for CCG population [27]**

Unplanned hospitalisation for asthma, diabetes and epilepsy in under 19s was almost 30% higher than the national average.

Ten per cent higher rate of mortality in under 75’s from cardiovascular disease.

Higher rates of hospital admissions and episodes due to falls than England (5.2 % in comparison to 3.3 % national average).

Higher death rate in hospital (62.6% compared to 54.5% national average).

Only 51.3% of people with a long term condition felt supported to manage their condition.

**Box 2: Limitations of local primary care services [27]**

1. **Fragmentation** due to disease-specific approach to long-term conditions management.
2. **Unsustainability in General Practice** due to model not responding to pressures facing general practice.
3. **Inequitable Service Provision** due to practices choosing which aspects of care they do and do not deliver.
4. **Inconsistency of offe**r prevented clarity about where general practice provision stops and community/secondary care provision begun.
5. **Monitoring burden** for general practice and CCG related to multiple disease-specific schemes with separate specifications.
6. **Limited transformation** as there was not a stretching vision of the role of primary care in delivering services currently delivered in secondary care.
7. **Inadequate workforce planning** through short-term contracts and insecure funding outside of an overarching framework.

The CCG therefore decided to introduce a new approach in which general practice and the wider community health services were more co-ordinated around practice populations. The ‘Aspiring to Clinical Excellence’ (ACE) programme sought ‘to make the paradigm shift needed to create a more integrated way of working led by strong primary care teams based around General Practices’ (p7) [27] (see **Box 3**). The programme had two levels – a *foundation level* which composed of the minimum standards expected from general practice and paid for through generic contract funding, and an *enhanced level* which would entail general practice delivering a greater breadth and depth of services by itself and/or in collaboration with other health and care organisations [27]. To understand how the enhanced level model could be implemented in practice, the CCG developed a twelve month pilot programme in which selected practices would develop new integrated approaches [29]. There were two sets of objectives for the pilots:

Programme Objectives 1: The delivery of key enablers to people with long term conditions in the community. These were diagnostic and treatment options that were largely provided in acute care settings. They included ECG testing and interpretation, spirometry, insulin initiation and phlebotomy. The practices could determine how best they delivered these enablers.

Programme Objectives 2: To develop new models of holistic care for those with complex mental and/or physical health needs. The practices could determine the local priorities in respect of population needs and opportunities to divert activity for acute hospital.

The pilots received additional funding of £10.50 per registered patient, and were selected competitively through an open call to all the member practices of the CCG. Interested pilots submitted a proposal which detailed how they would meet the required objectives and which demonstrated commitment from all of the involved practices. A panel made up of CCG Governing Body members and senior managers selected which pilots would receive funding. Various groupings of general practice were chosen to explore the benefits of alternative sizes and types of partnerships (see Table 1). Pilot leads were mostly GPs but also included practice managers (these individuals are employed by the GP partners to oversee administration of practices). The pilots were required to provide progress reports to the CCG and be willing to share learning with the wider practice membership. Each pilot also had to be represented at monthly action learning sets. These were an opportunity for peer support between pilots and for discussion with clinical and commissioning leads from the CCG. An action research based evaluation was contracted from the University of Birmingham to act as a critical friend to the pilot [30,31,32,33,34]. The focus was on the transformation process and learning for future change programmes. Mixed qualitative methods included observations of action learning set meetings, semi-structured interviews, and focus groups. Participants included Pioneer leads and the programme leads within the CCG (see Table 1). Data gathering was undertaken throughout the twelve months of the pilots and six-months post-programme. Emerging themes were shared periodically with the pilots and the CCG leads to generate discussion and test out validity of interpretation. The CCG also undertook a performance review of all pilots to establish their progress in achieving the programme objectives. The finding are based on the reflections of the programme participants, data collected by the CCG and the observations of the researchers. It is worth noting that due to the relatively short length of the programme an impact evaluation was not completed. The focus of the evaluation and the article is on the experience of this transformation process.

**Table 1:** Overview of the pilots.

Pilot	Number of Practices	Total Practice Population (approx)	Integration Innovation	Professional background of evaluation participants

A	12	53,000	Collaboration with acute sector diabetes team to provide development and clinical guidance to general practice rather than out-patient appointments.	GP
B	3	31,500	Use of a formal improvement methodology to reduce unplanned admissions and facilitate discharge of older people from acute hospital.	GP Nursing
C	5	13,000	No clear integration innovation identified.	GP Practice manager
D	1	11,000	Series of small scale pilots to address priority needs, including community chaplaincy, link worker to connect with local resources, transitional beds in nursing homes to facilitate early discharge and case management for those with multi-morbidities.	GP
E	9	65,000	During programme developed process through which paramedics could directly access GPs for discussion of patients at risk of admission.	GP
F	1 (but with 10 sites)	61,500	Initially proposed enhancing of multi-professional teams but then focussed on use of common IT system to support integration between different practices within organisation.	GP Practice manager

By the end of the programme all of the pilots had achieved Programme Objectives 1, i.e. the specified diagnostic and treatment activities. These were delivered through different arrangements that reflected local contexts and the professional judgements of the pilot leads. Some choose to provide the enablers through skilling up relevant staff members and purchasing the required equipment for each practice, others had hub and spoke models in which identified practice(s) would provide this service to patients across all the pilot grouping, and the remainder choose to buy one or more diagnostic support services from an external company. The successful achievement of these enablers meant that the CCG was subsequently able to decommission a number of diagnostic services from acute hospital services.

In relation to Objective 2, the pilots with concrete integration innovations at the time of application made more rapid progress than those with less concrete proposals (see Table 1). Pilot A developed a shared care arrangement between acute and primary care in relation to people with diabetes. This involved specialists from the hospital undertaking patient record based reviews with GPs who were less confident in acting as the lead clinician for patients with diabetes. Specialist doctors and nurse practitioners also participated in monthly team discussions with more confident GPs and practice nurses regarding specific individuals with complex diabetic needs. Pilot B engaged an external expert to provide training on improvement methodologies. They mapped out the journey of older people being subject to unplanned admissions to hospital through talking directly with older people and meeting with clinicians within the local emergency department. Pilot B then employed experienced community nurses as practice based case managers and introduced a bespoke notification process for patients who had been admitted to hospital. Pilot D tested out a smaller scale innovations with rapid review of their impact. If these were not seen as achievable or beneficial then they were not continued. These included the purchase of short term beds within a local care home and providing a contact GP beyond normal opening hours. Others, including the recruitment of a chaplain and community link worker were more promising and therefore refined and continued. Pilot E did not have a clear proposal for Objective 2 at the outset but subsequently developed a triaging process in which paramedics had access to a named GP to contact if they were considering admitting a patient to hospital.

For the first six months programme implementation proceeded as expected. Each pilot was represented at every learning set, although not always by the same individual(s). Initial learning sets were dominated by pilots seeking clarification with the commissioners regarding the award of the funding and what activities were expected. Eventually the pilots accepted that they did have autonomy to decide how to achieve the programme objectives. Thereafter the learning sets focussed on providing updates from pilot and associated learning, considered shared challenges and uncertainties, attempted to influence other stakeholders in the local health and care system, and reflected on the insights from the evaluation. The shared learning was the element that the pilot leads saw as crucial, but also something that they did not always actively participate in as individuals. There appeared to be complex dynamics in play in regards to the latter – a shyness in being seen to promote their own work, an unwillingness to share their innovations in case they were stolen by others, and an uncertainty that the work of others were relevant to their own context. The final learning set was seen by all those who attended as most fulfilling the potential for open and honest sharing. This may have been due to the relationships becoming stronger over the life of the learning set, or a sense that they had nothing to lose due to the pilot period coming to an end. Senior representatives from the local provider of community health services were invited to attend the learning sets on a regular basis. This enabled discussion of how community nursing services could be reconfigured to support the aspirations of the pilots.

As the programme progressed the CCG came under increasing pressure from other member practices who wanted to adopt the enhanced level and receive the related funding. This resulted in the wider roll out being commenced before the twelve month pilot phase was complete. The CCG also became concerned that the post-pilot practices may not be sufficiently committed to the transformation ethos and a directive commissioning approach would be required. This led to a more traditional contract being introduced in which the required activities were specified. Despite this, the innovations developed by pilots B, D and E continued and in some cases have been adopted by other practices. These have involved local engagement with other health and care partners. The attempts to use the learning set to more fundamentally influence the main local provider of community health services were less successful though due to other competing demands on their time and focus.

## Learning from the Programme

The ACE pilot initiative was not fully implemented as originally designed and did not achieve all of its objectives. It did though confirm that general practice was able to take greater responsibility for procedures previously delivered by acute care, and that with the right support and an enabling environment frontline primary care clinicians and practice managers could improve aspects of vertical and horizontal integration. The programme provides four main lessons regarding primary care transformation which will be of interest in other countries. Before listing these, it is worth noting just how different this programme felt to participants from previous reform initiatives. These had been based on a more traditional pay for performance model in which the commissioners tightly dictated the activities required to receive additional incentives. In contrast the ACE pilot programme was experienced by the pilots as a trust based model which gave flexibility for practices to respond to their local needs (see **Box 3**).

**Box 3: Participant perspectives**

“it was lovely to be given a pot of money to innovate, be creative, think outside the box…, we tried before, but it’s always been on the back of an envelope…. to be given time and resource is a lovely thing.” (Pioneer D).

“Previous years we’ll just do it because that’s somebody has decided and whilst we might get annoyed about it, you’d think well we’ve got to do the service to get paid as this is the spec.” (Pilot C).

“It’s a first dawning on me that we carried some gravitas, some weight. I’ve taken that idea and used it in other areas, because people do take notice when you get a name, or a bit of a reputation.” (Pilot E).

“Highs are sharing ideas and trying to solve problems and be creative in that [learning set] meeting. The lows are going round the houses and discussing things that you feel aren’t going to change anything…Different personalities create different discussions and you can’t predict how it’s going to go.” (Pilot D).

“This was an ideal opportunity for us to showcase the power of being a big practice, and what we’ve already got to deliver such things quickly.” (Pioneer F).

“It was supposed to be aspiring to clinical excellence, not aspiring to saving lots of money…Which is basically what we’re asked to do now, and our morale has gone down as a consequence.” (Pilot A).

“Previously we met very infrequently and had unstructured conversations that went off on a variety of tangents. Meeting weekly has been absolutely bedrock in making this work. We said, “For the first quarter we are going to do this. For the next quarter we’re going to do that”. We worked it out so that we understood the process before we leapt in and made a change.” (Pilot B).

“Initially we relied on a managerial structure and didn’t recognise the need for clinical leadership. We changed that momentum halfway through. We put clinical leaders to direct with managers, and used their shared skills more appropriately.” (Pilot F).

### Transformation requires shifts in established paradigms

The additional funding provided was used to purchase of diagnostic equipment, explore the impact of new posts, and free up time for key individuals involved in the change process. This practical support made important contribution but by itself would not have resulted in the expected transformation. This required the pilot leads to move beyond what they traditionally saw as their role within the local health and care system. Several commented how they had never viewed themselves as being leaders beyond the boundaries of their general practice. The programme provided them with both the impetus and the status to take a wider responsibility which resulted in more imaginative thinking and a belief that they could influence others. Linked to this was initial scepticism from the leads that the commissioner would be willing for pilots to try our new ways of working. Only by having the opportunity to repeatedly test out their uncertainties with the commissioning leads could they be convinced that a new approach would be tolerated. Similarly the commissioners had to develop an alternative mind set in which they would be less contract enforcers and more partners in uncertainty with the general practices. The programme therefore required both providers and purchasers to think differently about themselves and their relationships with other parts of the system.

### Learning opportunities need to be carefully designed

The programme was designed on the basis of the pilots sharing learning with each other as they progressed with their local innovations, and the pilots as a cohort sharing their learning with the wider membership of the CCG. The learning sets were pivotal in the first regard as they provided a safe space for pilots and commissioners to voice successes and frustrations, and consider ways of overcoming individual and collective challenges. The sets had a degree of structure with time for general updates, sharing of recent experiences, and one or two topics of shared interest. The sets though suffered from not having sufficient planning in how they would be facilitated. One of the pioneer leads took on this responsibility by default but did not have previous experience of undertaking such a facilitative role. This led to missed opportunities to shape the learning process within the meetings. A lack of consistency in who represented some pilots at each learning set potentially inhibited closer relationships being developed over time between the main individuals. The sharing of learning with the wider membership was not sufficiently planned and was essentially designed during implementation. Presentations were made at CCG member and public events with some articles in organisational newsletters but a more structured approach would have communicated insights more promptly and consistently for wider implementation. This would also have potentially appeased some of the critics in the wider membership who were not sure that the pilots were achieving anything new as such.

### Motivations can be nurtured on multiple levels

The leads reported several reasons to take on the additional responsibility of being a pilot. Most wanted to do something different to improve care for the patients in their locality but had not previously sufficient support or capacity. This motivation was related to a desire to *improve clinical care*. Alongside this, the national policy debates regarding the benefits of general practices working in partnerships made some leads believe that practice mergers could be dictated. This resulted in leads wanting to demonstrate the strength of their current arrangements or explore the potential of their choice of partner arrangements. This motivation was based on a desire for *organisational autonomy*. Being selected as a pilot gave the prestige of being portrayed as a local innovator in whom the CCG were willing to invest additional resources. Alongside the motivation of being *identified as an innovator*, was the concern that failure to deliver would receive harsh criticism from other practices who were not successful in their applications (*peer judgement*). The latter was a frequent source of discussion at the learning sets and added the sense amongst the pilots of being in collective endeavour. This was also felt by the commissioning leads in respect of the expectations of the CCG regarding the savings that would be delivered. Making initial progress consolidated the belief of pilots that change was possible and encouraged them to continue with their efforts (*encouragement of quick wins*). Conversely, if the innovations were not funded beyond the pilot stage then this was demotivating as the time and energy spent in the development was seen to have been lost. There are therefore multiple levels of motivation possible amongst participants in the same transformation programme. Recognising these and assessing regularly how these can be nurtured throughout is important in ensuring continued commitment.

### Inspiration needs to be accompanied by practical methodologies

The pilot leads entered into the programme with great aspirations for what could be achieved. All of them dedicated considerable time and energy in trying to take forward their ideas for how patient care could be more integrated. Their approach to delivering their transformations varied considerably both in relation to their change infrastructure and their improvement methodology. The pilots which invested in sufficiently freeing up clinicians and others to lead on their projects and a systematic improvement process had wider engagement amongst their pilot group practices and greater likelihood of sustainable impact. For example, Pilot B had dedicated lead GPs from each of the three practices who met every Wednesday morning to plan and review progress. They also introduced an on-line blog which shared developments and learning in real time with other staff members in their practices. Pilot F began with much of the work being undertaken principally by managers but found that clinicians sharing this responsibility led to more progress being made. Using an explicit methodology to guide improvement was also important. Pilot B enlisted the support of an external facilitator who provided training on improvement and guided them through a value based process. Pilot D used a simple Plan-Do-Study-Act approach for their mini-pilots which provided sufficient insights within three months for them to recognise which were promising and which should be discontinued. The programme as a whole struggled to provide meaningful activity and outcomes data for the individual pilots on a timely basis. This led to considerable frustration as it was not possible to provide a more objective comparison of the impacts of their alternative approaches. The overall emphasis on reducing activity in acute hospital care also meant that the benefits of innovations which may have had wider benefits and taken longer to achieve measurable outcomes were not fully recognised.

## Conclusion

Many of the components of a more integrated primary care system – putting patients at the centre of decision making, greater collaboration between professions, connecting with community assets, and developing new holistic roles – are known. The challenge that health and care systems face is how to change their current patterns of investments and established cultures to meaningfully and sustainably adopt these new ways of working. This will require not only changes in financial incentives, organisational partnerships and clinical pathways but also in the way that professionals and indeed patients and communities conceptualise their roles and contribution. Such transformational change is undoubtedly a challenging process which will involve a sophisticated mixture of levers and interventions. These must be tailored to the national and local contexts in which they are being implemented and respond to the individual and collective challenges and aspirations of participants. Sharing learning between programmes and systems will help us to collectively understand how we can increase the likelihood of transformations being achieved. This includes those elements that have not been as impactful as hoped, as well as those which have been relatively successful. The experience of this programme suggests that transformation requires a mixture of visionary inspiration and practical methodologies, designated senior and distributed frontline leadership, and structured opportunities to test and learn. It also highlights again the challenge of timely data, that sustainability can be conceptualised not only as the continuing of planned interventions but also in a legacy of aspiration and belief, and that with the right combination transformation in primary care is possible:

“To be given an opportunity to bring in innovative changes and actually be rewarded for it as a practice is a massive step forward…it’s like a new beginning. This is the true spirit of commissioning: ‘We’re treating you like grown-ups. Never mind the tick-boxes. Go and innovate and we’ll pay you to do it – properly’.” (Pioneer D)
